# Raised FGF-21 and Triglycerides Accompany Increased Energy Intake Driven by Protein Leverage in Lean, Healthy Individuals: A Randomised Trial

**DOI:** 10.1371/journal.pone.0161003

**Published:** 2016-08-18

**Authors:** Alison K. Gosby, Namson S. Lau, Charmaine S. Tam, Miguel A. Iglesias, Christopher D. Morrison, Ian D. Caterson, Jennie Brand-Miller, Arthur D. Conigrave, David Raubenheimer, Stephen J. Simpson

**Affiliations:** 1 Charles Perkins Centre D17, The University of Sydney, Sydney, New South Wales, Australia; 2 School of Life and Environmental Sciences, The University of Sydney, Sydney, New South Wales, Australia; 3 Boden Institute of Obesity, Nutrition, Exercise and Eating Disorders, The University of Sydney, Sydney, New South Wales, Australia; 4 School of Health Sciences, University of Tasmania, Darlinghurst Campus, Darlinghurst, New South Wales, Australia; 5 Pennington Biomedical Research Centre (PBRC), Baton Rouge, Louisiana, United States of America; 6 School of Veterinary Science, The University of Sydney, New South Wales, Australia; Pennington Biomedical Research Center, UNITED STATES

## Abstract

A dominant appetite for protein drives increased energy intake in humans when the proportion of protein in the diet is reduced down to approximately 10% of total energy. Compensatory feeding for protein is apparent over a 1–2 d period but the mechanisms driving this regulation are not fully understood. Fibroblast growth factor-21 (FGF-21) has been identified as a candidate protein signal as levels increase in the circulation when dietary protein is low. The aim of this randomised controlled trial was to assess whether changes in percent dietary protein over a 4 d *ad libitum* experimental period in lean, healthy participants influenced energy intake, metabolic health, circulating FGF-21 and appetite regulating hormones including ghrelin, glucagon like peptide-1 and cholecystokinin. Twenty-two lean, healthy participants were fed *ad libitum* diets containing 10, 15 and 25% protein, over three, 4 d controlled, in-house experimental periods. Reduced dietary protein intake from 25% to 10% over a period of 4 d was associated with 14% increased energy intake (p = 0.02) as previously reported, and a 6-fold increase in fasting circulating plasma FGF-21 levels (p<0.0001), a 1.5-fold increase in serum triglycerides (p<0.0001), and a 0.9-fold decrease in serum total cholesterol (p = 0.02). Serum HDL cholesterol was reduced with a reduction in dietary protein from 15% to 10% (p = 0.01) over 4 d but not from 25% to 10% (p = 0.1) and the change from baseline was not different between diets. Plasma fasting insulin levels following the 4 d study period were significantly lower following the 25% *ad libitum* study period compared to the 15% protein period (p = 0.014) but not the 10% protein period (p = 0.2). Variability in interstitial glucose during each study period increased with a decrease in dietary protein from 25% to 15% and 10% (p = 0.001 and p = 0.04, respectively). Ghrelin, glucagon-like peptide-1 and cholecystokinin were unchanged. Increases in energy intake, plasma FGF-21 and serum triglycerides were associated with reductions in percent dietary protein from 25% to 10% energy over a 4 d *ad libitum* in-house feeding period and may be important in regulation of dietary protein intake.

**Trial Registration:**

Australia New Zealand Clinical Trials Registry ACTRN12616000144415

## Introduction

In a range of species, from locusts to rodents and humans, total energy intakes increase with a fall in percent dietary protein down to a point where excessive protein dilution fails to elicit increased intake. This response reflects an underlying nutrient-specific appetite for protein and has been termed the protein leverage effect [1–3]. A dominant appetite for protein driving energy intake has also been proposed to contribute to development of the human obesity epidemic [2, 3]. A recent analysis of 38 published experimental trials [4] and results from population studies and large dietary trials [5–7] shows that protein drives energy intake most strongly in humans when dietary protein is between 10 and 20% but not when percent dietary protein falls to lower levels of 5% [4, 8, 9] or is elevated to levels exceeding 30% [4]. Together these data suggest that protein leverage operates within a range of values for percent dietary protein that reflect the usual range seen in human populations with food sufficiency.

The mechanisms that control protein intake are not well understood. In humans protein leverage is expressed over a 1–2 d period [10, 11]. A shorter period of satiety follows a low protein meal when compared to a higher protein meal [11, 12] and may be important in driving increased food and energy intake in the longer term by increasing the frequency of feeding episodes. Dietary studies in humans suggest that taste cues associated with protein in food, such as umami, elicit elevated activity in the brain regions associated with hedonic responses when protein is depleted [13]. It has been hypothesised that there may be a systemic hormonal ‘protein signal’ that responds specifically to low levels of dietary protein, which can override nutritional feedbacks arising from high levels of carbohydrate and/or fat intake to permit continuing energy intake so that target protein intakes can be attained [2, 4]. Recently, Fibroblast Growth Factor-21 (FGF-21) was described as a potential candidate for such a protein signal. Laeger *et al*. [14] reported that FGF-21 was elevated, in mice and in humans, during fixed energy feeding of a low protein diet (5% dietary protein) [15]. The elevation in FGF-21 levels observed in humans following an overnight fast suggests that a sustained effect of a low intake of dietary protein triggers FGF-21 release. Appetite regulating hormones like cholecystokinin (CCK), glucagon like peptide -1 (GLP-1) and ghrelin respond to feeding [16–19] and in some studies, but not all, the response varies with dietary macronutrient composition (reviewed in [20, 21]). However, the relationships between the fasting levels of these hormones and sustained changes in percent dietary protein have not been studied in detail.

The aim of this study was to assess the plasma levels of FGF-21 and known appetite-regulating hormones including the pro-appetite hormone ghrelin and satiety hormones GLP-1 and CCK and various metabolic variables in lean, healthy individuals to a change in the effect of percent dietary protein under *ad libitum* conditions in which dietary macronutrient composition was disguised [11]. We hypothesised that a reduction in percent protein would increase the level of serum FGF-21 and ghrelin and lower the levels of the satiety hormone GLP-1 and CCK.

## Methods

### Ethics statement

The study protocol (S1 File) was approved in March 2007 by Sydney South West Area Health Service (Royal Prince Alfred Hospital) Human Research Ethics Committee (Protocol No. X07-0044) and the University of Sydney Human Research Ethics Committee (Ref No. 10153) and is registered with the Australian and New Zealand Clinical Trial Registry (ACTRN12616000144415). The trial was registered after participant recruitment began. The authors confirm that all ongoing and related trials for this intervention are registered. The study is an experimental study to test the response to a changed macronutrient composition of the diet rather than to test a clinical dietary intervention.

### Study participants

**Fig 1:**
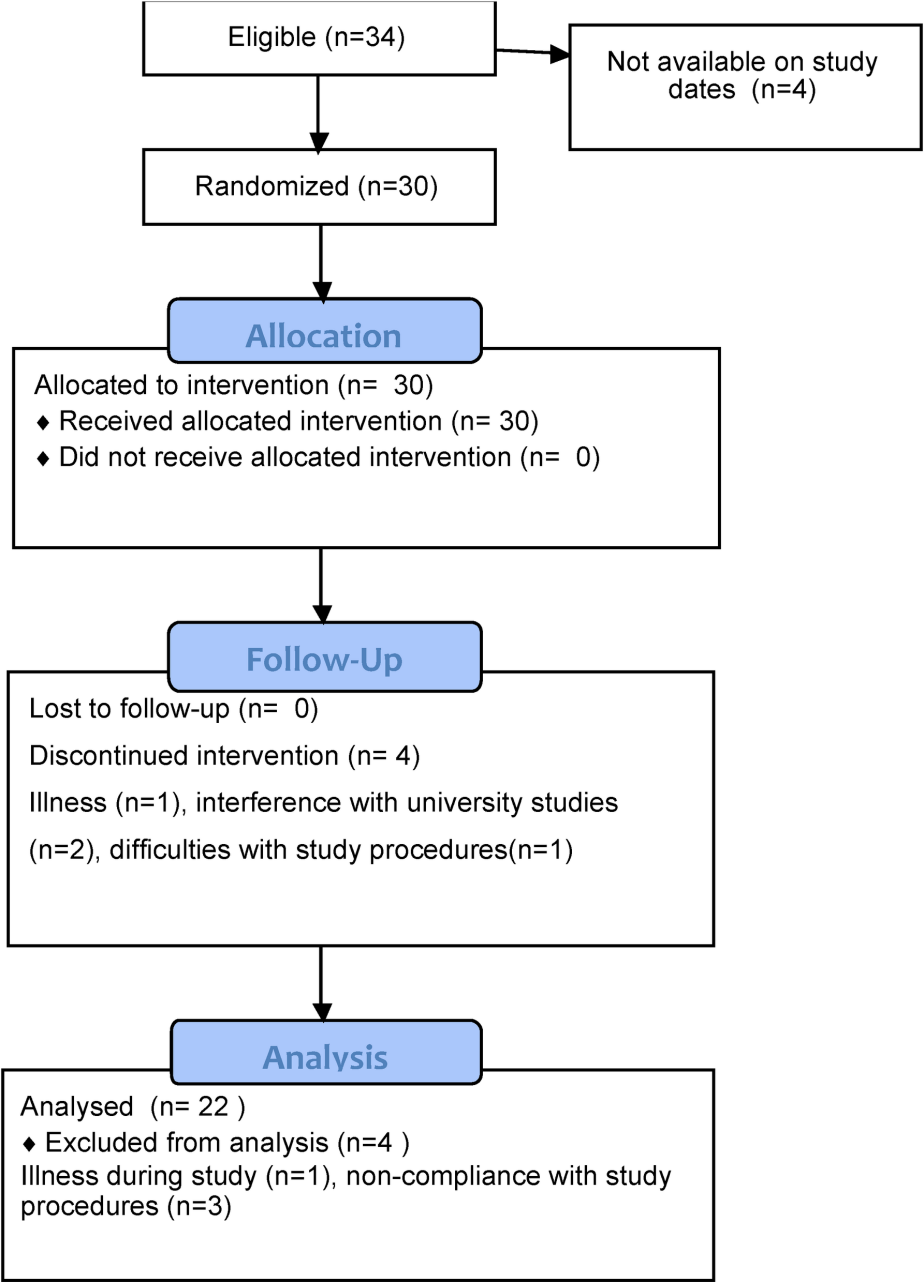
CONSORT 2010 flow diagram.

As previously described [11], lean, healthy (BMI:18–25 kg.m^-2^) male and female participants were recruited by advertising through casual employment sites at five universities within the Sydney region during October 2007—Dec 2009. All patient follow-up was completed by February 2011. Fig 1 presents the CONSORT 2010 flow diagram. 22 females and 12 males were eligible, indicated their willingness to undertake the trial and completed initial investigation day measures. From these, 20 females and 10 males commenced the trial. Subsequently, three females chose to discontinue due to interference with university studies, illness or difficulties with blood collection. One male was excluded after commencement following a diagnosis of hyperthyroidism. Overall, 17 lean female and 9 lean male participants completed the trial. On completion, four participants were excluded from the data analysis for reasons including gastrointestinal upset during one of the study weeks (n = 1 female) and failure of one sub-group to comply with study procedures (n = 3 males, consumed one another’s food). 16 lean female and 6 lean male participants were included in the final data analysis. Exclusion criteria were diabetes, high blood pressure, gastrointestinal problems, asthma, eczema or hay fever, chronic medical conditions, anaemia, allergies or strong dislikes to any study foods, smoking, following a weight reducing diet within the 3 months prior to the screening interview, pregnancy and breastfeeding. Participants completed the EAT-26 questionnaire and were excluded if they had a history of eating disorders or irregular eating habits. Vegetarians and vegans were excluded to aid in preparation of the treatment foods. All participants were given detailed verbal and written information regarding the purpose of the trial and the study procedures enabling written informed consent.

### Dietary protocol

The full details of the dietary manipulation have been published previously [11, 22]. Briefly, recipes were modified to contain 10, 15 or 25% energy as protein. Carbohydrate was adjusted to be 60, 55 or 45% energy and dietary fat was kept constant at 30%. The final *ad libitum* menus for each of the three 4 d study periods were matched for energy density, palatability and variety [11]. The diets were *ad libitum* and participants were free to eat at anytime of the day. The final menus and the total nutrients available to each participant in this study have been published previously [11]. Habitual intakes and *ad libitum* intakes in response to the 10%, 15% and 25% protein diets consumed by each participant in this study are also reported elsewhere [11].

### Study design

The details of the study design and participants are presented here (S1 File) and in Gosby et al. [11]. Following a screening interview each participant was allocated to a group of participants who were available to complete the trial on the same 3, 4 d periods of in-house dietary manipulation at the Woolcock Institute Sleep Study Centre, Glebe, NSW 2037, Australia. Once the subjects in each group were finalised the group was then randomly allocated an order of intervention by the study coordinator. Allocation of order was concealed by randomly selecting a sequence from an envelope. All possible sequences of interventions were printed the same number of times and placed in an envelope prior to the start of the study by the study coordinator. Participants were blinded to the sequence of interventions. It was estimated that 20 subjects would be required to detect a difference in ad libitum energy intake between dietary treatments. The sample size calculation was based on energy intakes from a previous *ad libitum* study where a 4.5MJ change in energy intake was measured in response to a reduction in percent dietary protein from 15% to 8% with a standard deviation of differences within pairs of approximately 1.2MJ. Using a paired, two-tailed design a difference of 0.8MJ, with a power of 80% and alpha of 0.05 could be detected with a sample size of 20.

Each study period was separated by at least 1 week. Participants arrived fasted on d 1 and were fasted from 10pm on d 4 of each 4 d *ad libitum* period. By the end of the experiment each group had undergone 4 continuous days on each of the 10, 15 and 25% dietary protein menus [11]. Participants were taken for a 1 hr walk on each of the study days.

### Continuous glucose monitoring

The Continuous Glucose Monitoring System Gold (CGMS) (Medtronic, CA91325, USA) was used to collect continuous glucose measurements during each 4 d experimental period. The CGMS was fitted on d 2 of each experimental period. Participants were given instructions to calibrate the monitor before breakfast, lunch, dinner and prior to going to bed each day. The calibration was performed by entering the contemporaneous glucose reading obtained from a finger prick sample (Accu-Chek Performa, Roche, USA). If the monitor did not calibrate participants were instructed to inform and get assistance from the study coordinator. Participants were asked to enter meal, snack and activity events into the continuous glucose monitor. The continuous glucose monitor was worn until waking on d 5 at which point a last calibration was performed and the monitor removed from the participant. The continuous glucose monitor recorded readings every 5 minutes following initialisation.

### Continuous glucose monitoring data analysis

To determine if there was an effect of percent dietary protein on the glucose profiles during d 3 and d 4 of *ad libitum* feeding of the 10%, 15% and 25% protein study periods the 24 h glucose data from the continuous glucose monitors on d 3 and 4 was used to calculate the average daily glucose, area under the curve for glucose and variance of glucose values during each 24-h period. The variance was determined by calculating the standard deviation of the blood glucose rate of change. The blood glucose rate of change (mg/dl/min) evaluates the dynamics of blood glucose fluctuations over 15-min periods [23]. A larger variation in the blood glucose rate of change indicates a more rapid and pronounced blood glucose fluctuation. Validation studies indicated that the Minimed CGMS is adequate for testing the glucose response to foods [24, 25].

### Sample analysis

Serum and plasma samples were taken from fasting participants on the morning of d 1 and d 5 of each study period. Samples were stored on ice and transported to Sydney South West Pathology Service, Royal Prince Alfred Hospital, a NATA/RCPA accredited laboratory or stored at -80°C for subsequent analysis of glucose, serum triglycerides and total and HDL cholesterol. Plasma FGF-21 was measured using the Human FGF-21 Quantikine ELISA Kit (DF2100) by R&D systems. This assay achieves a sensitivity of 8.69 pg/mL and has a coefficient of variation of 2.9–3.9% (intra-assay) and 5.2–10.9% (inter-assay) over a range of 31.3–2000 pg/mL. The GHRT-89HK Total Ghrelin RIA kit (Millipore Corp, Billerica, MA, USA) was used to measure total ghrelin in stored plasma. This assay achieves a sensitivity of 27.6 pmol/L, the coefficient of variation was 3.3–10% (intra-assay) and 14.7–17.8% (inter-assay) for concentrations of total ghrelin over the range 296.7–889.9 pmol/L. Plasma GLP-1 was measured using the Millipore GLP-1A-35HK kit (Millipore Corp, Billerica, MA, USA). DPP-IV inhibitor was added to the collection tubes. The assay achieved a sensitivity of 3pmol/L, the intra-assay coefficient of variation was 27–30% (intra-assay) and 12–34% (inter-assay) for concentrations of 14–40 pmol/L. Insulin concentrations of 42–325 pmol/L, the intra-assay coefficient of variation was 2.2–4.4% and the inter-assay co-efficient of variation 2.9–6% with the measured recovery after the addition of known concentrations of insulin into serum from 93–100%.

### Statistical analysis of experimental data

All data analysis and graphics were performed using R software (R version 3.2.3 [26]). The data are expressed as means ± SEM unless otherwise specified. All data were checked for normality using the Shapiro-Wilk test. Log transformation was used to normalise skewed data for body mass, diastolic blood pressure, triglycerides, total cholesterol, HDL cholesterol, ghrelin, GLP-1, CCK and for mean glucose, area under the curve and glucose variability data collected from CGMS. Square root transformation was used for FGF-21 data. Subjects with missing values for an analysis were removed from the analysis.

One-way within subject ANOVA was used to test for effect of percent dietary protein on d 5 circulating levels of insulin (n = 10, 54% missing), ghrelin (n = 19, 14% missing), CCK (n = 18, 18% missing) and GLP-1 (n = 19, 14% missing) following each 4 d study period. These data were checked for sphericity using Mauchlys sphericity test (*Multcomp* package [27]). If the sphericity assumption was violated, the Greenhouse-Geiser correction was applied to the F- and p-values for effect of percent dietary protein. Post-hoc analysis was performed with pair-wise comparisons using the Bonferroni correction for multiple comparisons. p < 0.05 was used to determine significance.

Mixed model linear regression was performed using the lme 4 [28] and lmerTest [29] packages in R to test for significant effects of percent dietary protein and for differences in the change from d 1 to d 5 between the dietary treatments. Percent dietary protein (10%, 15% and 25%) and time (d 1 and d 5) were used as fixed effects and participants as random effects. Day 1 and d 5 body mass (n = 22), systolic (n = 21, 5% missing) and diastolic (n = 21, 5% missing) blood pressure, fasting glucose (n = 18, 18% missing), FGF-21 (n = 11, 50% missing), triglycerides (n = 19, 14% missing), total cholesterol (n = 19, 5% missing) and HDL-cholesterol (n = 16, 27% missing) were collected prior to and after each 4 d dietary treatment. Mixed model linear regression was also used to assess the effect of percent dietary protein on d 3 and d 4 average daily glucose, area under the curve for glucose and standard deviation for blood glucose rate of change calculated from continuous glucose monitor data collected over each 4 d study period (n = 19, 14% missing). Each linear mixed model was checked to confirm that the errors had relatively constant variance, were independent and normally distributed. Mixed model linear regression analysis is presented as estimates, upper and lower confidence intervals (CI) and the p-value. p < 0.05 was used to determine significance.

Simple linear regression was used to test relationships between d 5 triglycerides with d 4 average daily glucose, d 4 area under the curve for glucose, d 4 glucose variability and d 4 energy intake.

## Results

### Subject characteristics

17 lean female and 9 lean male participants aged 24 ± 1 years (mean ± SEM; range 18–51) and BMI of 21.8 ± 0.4 (mean ± SEM; range 18–25.5) kg.m^-2^ completed the trial [11]. Participants’ habitual average intake was estimated to be 10.1 ± 0.6 MJ.d^-1^ and the macronutrient composition of the diet was 18.4 ± 0.7% protein, 47.2 ± 1.2%, carbohydrate and 34.4 ± 1.0% fat with an average protein intake of 78g.d^-1^[11]. The baseline measures and characteristics for males and females are presented in Table 1.

**Table 1:** Baseline characteristics of the participants.

	Female	Male
N	17	9
age (years)	25.2 ± 1.9	23.3 ± 0.8
body mass (kg)	55.5 ± 1.6	69.9 ± 3.0
height (m)	1.6 ± 0.02	1.8 ± 0.03
BMI (kg.m^-2^)	21.7 ± 0.5	22.1 ± 0.6
energy intake (MJ.d^-1^)	7.8 ± 0.2	10.1 ± 0.5
protein (% of energy)	17.6 ± 0.5	18.3 ± 0.9
carbohydrate (% of energy)	50.9 ± 0.7	49.8 ± 1.4
fat (% of energy)	31.4 ± 0.5	31.9 ± 0.9

### Circulating levels of plasma FGF-21 responded to percent dietary protein

As previously reported [11], over the 4 d ad libitum periods of the study participants increased their total daily energy intake by 14% when percent dietary protein was reduced from 15% to 10% but did not change total energy intake when percent dietary protein increased from 15% to 25%. Mixed model linear regression analysis (Table 2) was used to determine differences in fasting levels of FGF-21 between the diet groups on the morning of d 5 following the 4 day ad libitum study period and the difference in the change between d 5 to d 1 between diet groups. On the morning of d 5 plasma FGF-21 levels were 1.6-fold greater after the period of the 10% protein diet than following 15% dietary protein (p = 0.17) and 6-fold greater than levels following 25% dietary protein (p<0.0001) (Table 2). The change between d 5 and d 1 values was greater in response to the 10% protein diet in comparison to the 25% protein diet (p = 0.007, respectively) but not the 15% protein diet (p = 0.2) (Table 2).

**Table 2:** Mixed model linear regression estimates for circulating fasting serum FGF-21 levels (pg/mL) prior to and following each experimental period.

Model factor	estimate (CI lower, upper)	P-value*
Fixed effects		
Intercept (10% protein, d 5)	300 (206, 394)	
Slope (10% protein, d 1)	-153 (-260, -46)	0.02
Slope (15% protein, d 5)	-110 (-213, -6)	0.2
Slope (25% protein, d 5)	-251.64 (-355, -148)	<0.0001
Slope (15% protein d 1 to d 5)	131 (-15, 278)	0.2
Slope (25% protein d 1 to d 5)	194 (47, 340)	0.007
Intercept (15% protein, d 5)	190.0 (96, 284)	
Slope (15% protein, d 1)	-21.45 (-129, 86)	0.4
Slope (25% protein, d 5)	-142.0 (6, 213)	0.0006
Slope (25% protein d 1 to d 5)	62.18 (-84, 209)	0.1
Intercept (25% protein, d 5)	48.0 (-46, 142)	
Slope (25% protein, d 1)	40.73 (-66, 148)	0.2

*Square root transformation was used to normalise data for mixed model linear regression, untransformed data is presented.

### Plasma levels of fasting ghrelin, GLP-1 and CCK did not change with percent dietary protein

**Fig 2:**
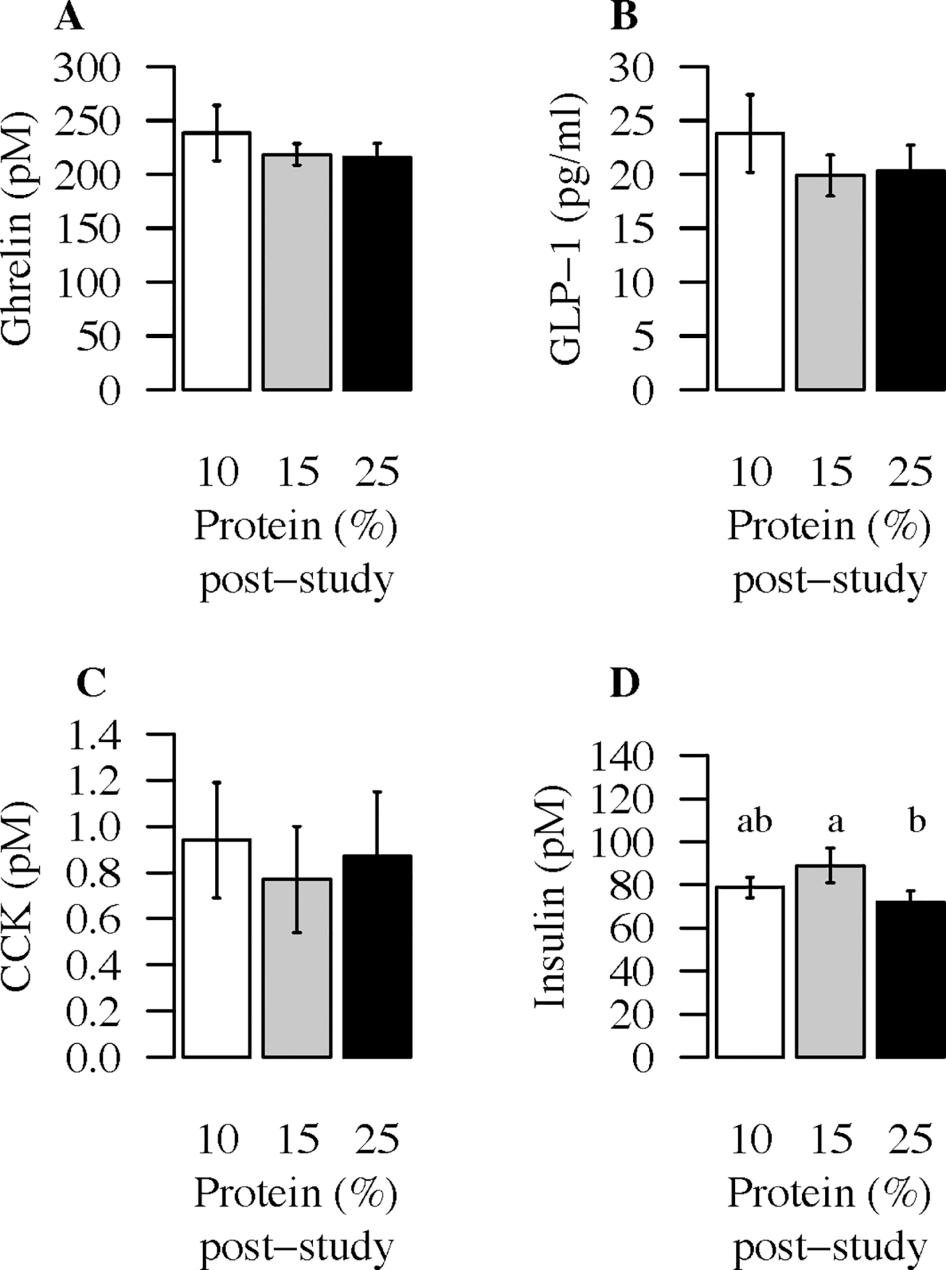
Plasma levels of fasting ghrelin, GLP-1, CCK and insulin in response to dietary treatments. Plasma (A) ghrelin (pM), (B) GLP-1 (pM) (C) CCK (pM) and insulin (pM) (D) on d 5 following each 4 d *ad libitum* 10% (white), 15% (grey) and 25% (black) protein study periods. A one-way within subject ANOVA was used to test for differences between dietary interventions for ghrelin, GLP-1, CCK and insulin. D 5 ghrelin, GLP-1 and CCK did not differ between diets, p>0.05. D 5 insulin concentrations were different between the diets (F_(2,38)_ = 6.0, p = 0.006). Different letters indicate significant differences between the groups, (d5 insulin: 10% vs 25% protein p = 0.2, 15% vs 25% p = 0.014).

One way ANOVA did not show any significant effect of percent dietary protein on d 5 fasting plasma levels of ghrelin (F_(2,36)_ = 0.5, p = 0.6), GLP-1 (F_(2,36)_ = 0.3, p = 0.7), or CCK (F_(2,34)_ = 1.9, p = 0.2) (Fig 2A–2C).

### Effect of percent dietary protein on body mass and blood pressure after 4 d of *ad libitum* feeding

Mixed model linear regression analysis (Table 3) was used to determine differences in body mass, and blood pressure between the different diet groups on d 5 and to assess whether there were any differences in the changes between d 1 and d 5 between diet groups. Day 5 body mass and systolic blood pressure was not significantly different following the 10, 15 and 25% protein diets (Table 3). However, d 5 diastolic blood pressure was reduced in comparison to 10% and 25% dietary protein (Table 3).

**Table 3:** Fixed effects estimates from mixed model linear regression analysis for body mass, systolic and diastolic BP measured on d 1 and d 5 of each 10, 15 and 25% protein study period.

Model factor	Body mass (kg)		Systolic BP (mmHg)		Diastolic BP (mmHg)	
	estimate	p-value*	estimate	p-value	estimate	p-value*
Intercept (10% protein, d 5)	59.4 (55.5, 63.3)		102.5 (99.3,105.8)		66.3 (63.9, 68.8)	
Slope (10% protein, d 1)	0.6 (0.1, 1.0)	0.016	-0.95 (-4.16,2.27)	0.56	-1.1 (-3.4, 1.1)	0.3
Slope (15% protein, d 5)	-0.25 (-0.7, 0.2)	0.3	-1.48 (-4.59,1.64)	0.4	-2.7 (-4.8, -0.6)	0.01
Slope (25% protein, d 5)	-0.2 (-0.6, 0.3)	0.5	-0.3 (-3.45,2.79)	0.8	-0.3 (-2.4, 1.8)	0.7
Slope (15% protein d 1 to d 5)	-0.5 (-1.1, 0.1)	0.08	-0.14 (-4.55,4.27)	0.95	3.6 (0.7, 6.6)	0.02
Slope (25% protein d 1 to d 5)	-0.4 (-1.0, 0.2)	0.2	-2.67 (7.08,1.74)	0.2	-1.4 (-4.4, 1.5)	0.3
Intercept (15% protein, d 5)	59.2 (55.3, 63.1)		101.1 (97.9,104.3)		63.6 (61.2, 66.1)	
Slope (15% protein, d 1)	0.02 (-0.4, 0.5)	0.92	-1.1 (-4.3,2.1)	0.5	2.5 (0.3, 4.7)	0.04
Slope (25% protein, d 5)	0.1 (-0.3, 0.5)	0.73	1.14 (-1.98,4.26)	0.5	2.4 (0.3, 4.5)	0.026
Slope (25% protein d 1 to d 5)	0.2 (-0.4, 0.8)	0.6	-2.5 (-6.93,1.89)	0.3	-5.0 (-8.0, 2.1)	0.001
Intercept (25% protein, d 5)	59.2 (55.4, 63.2)		102.2 (99.0,104.5)		66.0 (63.5, 68.5)	
Slope (25% protein, d 1)	0.2 (-0.3, 0.6)	0.4	-3.6 (-6.82,-0.41)	0.03	-2.6 (-4.8, -0.3)	0.019

*Log transformation was used to normalise data for mixed model linear regression, untransformed data is presented.

### Glucose control

Linear mixed model regression analysis showed that fasting glucose did not differ between the 10%, 15% or 25% protein diets (F_(2,34)_ = 1.29, p = 0.3) (Table 4). Using a within subject one-way ANOVA, however, d 5 fasting insulin concentrations were different between the diets (F_(2,38)_ = 6.0, p = 0.006). On the morning of d 5 following the 4 d study period fasting insulin levels were significantly lower following the 25% *ad libitum* study period compared to the 15% protein period (p = 0.014) but not the 10% protein period (p = 0.2) (Fig 2D).

**Table 4:** Fixed effects estimates from mixed model linear regression analysis for glucose control in response to 10, 15 and 25% protein diets.

Model factor	Fasting glucose (mM)	Mean glucose* (mM)	Glucose AUC*	SDROC	
	estimate	estimate	estimate	estimate	p-value*
Intercept (10% protein, d 4)	4.6 (4.5, 4.8)	5.57 (5.32, 5.82)	8009 (7622, 8397)	0.0215 (0.018,0.025)	
Slope (10% protein, d 3)	-0.02 (-0.22, 0.18)	-0.04 (-0.24, 0.17)	-119 (-437, 198)	-0.001 (-0.005,0.002)	0.4
Slope (15% protein, d 4)	0.06 (-0.11, 0.23)	-0.22 (-0.43, -0.02)	-224 (-546, 98)	-0.002 (-0.0016, 0.006)	0.2
Slope (25% protein, d 4)	0.08 (-0.09, 0.25)	-0.12 (-0.32, 0.09)	-172 (-494, 150)	-0.004 (-0.007, -0.0004)	0.04
Slope (15% protein d 4 to d 3)	0.07 (-0.17, 0.31)	0.04 (-0.25, 0.33)	97 (-339, 533)	-0.001(-0.006,0.004)	0.8
Slope (25% protein d 4 to d 3)	0.01 (-0.23, 0.25)	-0.08 (-0.37, 0.21)	-15 (-451, 421)	0.001 (-0.004, 0.006)	0.8
Intercept (15% protein, d 4)	4.70 (4.51, 4.89)	5.35 (5.10, 5.60)	7786 (-7398, 8173)	0.023 (0.020,0.026)	
Slope (15% protein, d 3)	0.06 (-0.14, 0.26)	0.01 (-0.20, 0.21)	-22 (-340, 295)	-0.002 (-0.006, 0.001)	0.3
Slope (25% protein, d 4)	0.02 (-0.15, 0.19)	0.11 (-0.1, 0.31)	51 (-271, 373)	-0.006 (-0.010, -0.002)	0.001
Slope (25% protein d 4 to d 3)	-0.06 (-0.30, 0.18)	-0.12 (-0.41, 0.17)	-112 (-548, 324)	0.002 (-0.003, 0.007)	0.6
Intercept (25% protein, d 4)	4.72 (4.54, 4.91)	5.45 (5.20, 5.70)	7837 (7450, 8224)	0.017 (0.014, 0.02)	
Slope (25% protein, d 3)	-0.01(-0.18, 0.30)	-0.12 (-0.32, 0.09)	-134 (-451, 183)	-0.0004 (-0.004, 0.003)	0.7

*Log transformation was used to normalise data for mixed model linear regression, untransformed data is presented. P-value is for analysis of Std deviation rate of change (SDROC).

Linear mixed model regression analysis was used to analyse the effects of percent dietary protein on continuous glucose monitoring results collected over d 3 and d 4 of each experimental period. The analysis suggested that average glucose and area under the curve (AUC) for glucose measured over d 3 and d 4 of each 4 d *ad libitum* study period did not change in response to percent dietary protein (Table 4). However, by study d 4, the standard deviation for blood glucose rate of change (SDROC), a measure of variability in interstitial glucose level, decreased as percent dietary protein increased from 10% to 25%. Then, glucose variability was reduced for the 25% protein diet in comparison to both the 10% (p = 0.04) and the 15% protein diets (p = 0.001) (Table 4).

### Triglycerides increased and total cholesterol decreased with a reduction in the proportion of protein in the diet

Table 5 presents a summary of the linear mixed model regression analysis of the effect of percent dietary protein on fasting serum levels of triglycerides, total cholesterol and HDL cholesterol measured on the morning of d 1 and d 5 prior to and following each 4 d ad libitum period. Day 5 triglyceride levels were 1.2-fold and 1.5-fold greater on the 10% protein diet than 15% protein diet (p = 0.04) and 25% protein diet (p<0.0001) (Table 5). Consistent with this, the change in the fasting serum triglycerides from d 1 to d 5 following 4 d of 10% dietary protein was greater than the change following 4 d of 15% (p = 0.08) and 25% dietary protein (p = 0.0005). Simple linear regression showed that an increased serum triglyceride level was associated with the dilution of percent dietary protein (p = 0.03), increased d 4 mean daily glucose level (p = 0.002) and increased d 4 area under the curve for glucose (p = 0.0009) but not with increased d 4 energy intake (11) (p = 0.3) or increased d 4 glucose variability (p = 0.9). D 5 total cholesterol level was 0.9-fold for 10% protein when compared to either 15% (p = 0.02) or 25% dietary protein (p = 0.03) (Table 5). The change from d 1 to d 5 following 10% dietary protein was significantly different from the change from d 1 following 15% and 25% dietary protein (p = 0.02 and p = 0.05, respectively). D 5 HDL-cholesterol levels were lower following 10% dietary protein in comparison to 15% (p < 0.01) but not 25% (p = 0.1) protein diets. The changes from d 1 to d 5 were not different between diets (Table 5). Despite the changes measured all serum lipid levels remained within their normal reference ranges during the course of these experiments.

**Table 5:** Fixed effects estimates from mixed model linear regression analysis for fasting serum lipids in response to percent dietary protein.

Model factor	Triglycerides (mM)		Cholesterol (mM)		HDL (mM)	
	Estimate	p-value*	estimate	p-value*	estimate	p-value*
Intercept (10% protein, d 5)	1.79 (1.42, 2.16)		4.26 (3.79, 4.73)		1.27 (1.15, 1.38)	
Slope (10% protein, d 1)	-1.02 (-1.35, -0.68)	<0.0001	0.06 (-0.16, 0.30)	0.46	0.19 (0.10, 0.28)	<0.0001
Slope (15% protein, d 5)	-0.34 (-0.59, -0.08)	0.04	0.27 (0.05, 0.50)	0.017	0.11 (0.02, 0.19)	0.0099
Slope (25% protein, d 5)	-0.64 (-0.90, -0.38)	<0.0001	0.24 (0.02, 0.47)	0.03	0.06 (-0.03, 0.15)	0.151
Slope (15% protein d 5 to d 1)	0.37 (0.01, 0.74)	0.078	-0.35 (-0.67, -0.03)	0.02	-0.09 (-0.22, 0.03)	0.091
Slope (25% protein d 1 to d 5)	0.68 (0.32, 1.05)	0.0005	-0.27 (-0.59, 0.05)	0.05	0.0006 (-0.12, 0.12)	0.94
Intercept (15% protein, d 5)	1.45 (1.08, 1.82)		4.54 (4.07, 5.0)		1.37 (1.26, 1.49)	
Slope (15% protein, d 1)	-0.64 (-0.98, -0.31)	<0.0001	-0.28 (-0.52, -0.05)	0.01	0.1 (0.01, 0.18)	0.04
Slope (25% protein, d 5)	-0.34 (-0.56, -0.05)	0.02	-0.03 (-0.26, 0.19)	0.88	-0.05 (-0.14, 0.04)	0.23
Slope (25% protein d 1 to d 5)	0.31 (-0.05, 0.67)	0.07	0.08 (-0.23, 0.40)	0.68	0.09 (-0.03, 0.22)	0.1
Intercept (25% protein, d 5)	1.15 (0.76, 1.52)		4.51 (4.04, 4.97)	<0.0001	1.32 (1.21, 1.44)	
Slope (25% protein, d 1)	-0.33 (-0.67, 0.05)	0.0004	-0.2 (-0.43, 0.03)	0.046	0.19 (0.10, 0.28)	<0.0001

*Log transformation was used to normalise for mixed model linear regression, untransformed data is presented.

## Discussion

In this study, the fasting plasma level of FGF-21 was higher following 4 d *ad libitum* intake of a reduced percent protein diet (10%), consistent with published work in mice and in humans [14]. Furthermore, studies in FGF-21 knockout mice indicate that FGF-21 is required for the increased energy intake that occurs on low percent protein diets [14]. In the present study, protein concentration in the diet was varied by substitution with carbohydrate with fat kept constant. Therefore the elevated serum FGF-21 levels and energy intakes observed in subjects on the 10% protein diets also occurred in the context of higher carbohydrate intakes (see also [14,30]).

Rodents have separate appetites for both protein and carbohydrate with protein dominating (to a somewhat lesser extent in mice than rats on the basis of the literature) [31, 32]. If the regulations of protein and carbohydrate intakes are comparable in humans, as indicated by other clinical trial and population data [5–7], the increase in energy intake on the 10% protein diet is predominantly due to limited protein availability rather than high levels of carbohydrate [11]. However, systematically testing the interactions between protein, fat and carbohydrate with other dietary factors such as carbohydrate quality, fiber content, amino acid and fatty acid composition, and dietary energy density on energy intake is required to unravel relative significance of each of these nutritional factors on energy intake and satiety.

The plasma level of FGF-21 was increased in mice fed a protein restricted-normal energy diet (10% dietary protein) in comparison to mice fed a protein restricted-low energy diet (20% protein) [14]. In healthy men and women plasma FGF-21 levels increased 1.7-fold above baseline [14] in response to a 28 d overfeeding protocol using a 5% protein diet [15] but did not change from baseline in response to overfeeding using a 15% protein dietary protocol [15]. In the current study plasma FGF-21 levels and energy intakes were increased following 4 d reduction in dietary protein from 25% to 10% using an approach in which dietary fat was held constant. Vienberg *et al*. also observed substantial increases in serum FGF-21 levels when dietary protein fell from 15 to 7.5% by increasing dietary fat content [33]. In contrast, a delayed increase in serum FGF-21 level was observed after a 9 d fast in humans [34]. In a recent study by Dushay *et al*. serum levels of FGF-21 were increased at 60 min in response to a dietary fructose load [35] indicating that increased carbohydrate intake on the 10% protein diet may also contribute to increased secretions of FGF-21. Coincident increases in serum alanine aminotransferase (ALT) and aspartate aminotransferase (AST) levels suggest that liver stress occurred under the fasting conditions [34]. ALT also increases on low protein, high energy diets used to determine protein requirements in nitrogen balance studies [36]. These findings raise the possibility that hepatic stress and catabolism indicative of liver inflammation or damage may be related to elevated serum FGF-21 levels.

Once stimulated, FGF-21 secretion from the liver may act on the hypothalamus to drive increased energy intake [37]. It has been suggested that essential amino acid deficiency may be detected by an alternative pathway involving the protein kinase General Control Nonderepressible 2 (GCN2) in the anterior piriform cortex (APC) and consequent phosphorylation of eukaryotic initiation factor 2α (eIF2α) which stimulates FGF-21 but also prevents further consumption of foods deficient in essential amino acids [37] and this can be reversed by the addition of methionine to a low carbohydrate ketogenic diet which may indicate an amino acid specific response [38]. Functional magnetic resonance imaging has been used to show an association between protein status and brain responses in reward regions to protein cues such as umami taste, protein depleted participants showed increased responses in comparison to protein replete participants [13], this may enable aversion to low protein foods in a protein-depleted state. In the current study, where diet derived amino acids were reduced the 14% increase in energy intake during the 10% protein period was largely achieved by preferential selection of savoury-flavoured scones and muffins between meals [11]. The differential response to low total levels of amino acids versus selective amino acid deficiency may partly explain why protein intakes are not fully stabilised across a range of values for percent dietary protein [4, 11]. For example, Martens *et al*. [8, 9, 39] found that food and energy intakes did not continue to increase when dietary protein was reduced to 5% protein, and energy intake did not fall in the current study at 25% protein [11]. A recent clinical trial in which disguised diets were offered to subjects for a period of 5 d found a monotonic decline in food intake as percent protein rose from 10, 15 to 25%, but even then total intakes of protein fell as percent dietary protein fell indicating that protein leverage was incomplete [40]. Such incomplete protein leverage is a widespread feature among animals, including mice. Among mammals, spider monkeys come the closest to complete expression of protein leverage by maintaining protein intakes almost constant as percent dietary protein of the diet changes in response to season from approximately 4% to 20% protein [41]. Food choice by taste preference may be disrupted when processed foods that are savoury in taste (eg. by addition of sodium glutamate) have low protein and high fat or carbohydrate content [42].

Elevated plasma levels of FGF-21 have been linked with improvements in metabolic health indicators [43–45]. The present study was relatively short in duration and cannot be used to comment on longer term effects of percent dietary protein and FGF-21 on metabolic health. Also, in contrast to habitual diets all foods in each dietary treatment were of a fixed macronutrient composition and it is not known whether this difference influences metabolic health. Deleterious changes in lipids such as increased serum triglyceride levels followed reductions in percent dietary protein whereas total cholesterol was reduced on 10% relative to 15% and 25% dietary protein. Indicators of glucose control (glucose variability and insulin) improved on the 25% relative to 15% and 10% dietary protein however the change in glucose variability was relatively small and may not be physiologically significant in this lean healthy population. The increase in serum triglycerides in response to the 10% protein diet could be detrimental to health if continued over longer periods. Dietary recommendations to increase carbohydrate by increasing complex carbohydrates rather than refined sources may reduce these detrimental effects on triglycerides and HDL-cholesterol [46] observed in the case of a high carbohydrate, low fat diet [47]. Longer-term dietary studies in mice and humans show increased longevity and better metabolic health on low protein, high carbohydrate diets [48–50]. The work by Solon-Biet *et al*. measures the metabolic response over a much larger range of dietary macronutrient compositions highlighting the importance of higher carbohydrate and lower levels of fat in improvement of cardio-metabolic disease and lifespan measures [48].

The current study did not provide any evidence for involvement of ghrelin, GLP-1 and CCK in percent dietary protein dependent changes in energy intake. A single, very high percent dietary protein meal (58.1% vs a control meal of 19.3% and 71% vs a control meal of 1% protein) enhanced post-meal suppression of ghrelin [51, 52] but had no effect when changes in percent dietary protein more closely resembled those of a normal balanced diet (ie. 35% vs 20% and 25% vs 10% dietary protein) [53, 54]. Secretion of GLP-1 may be stimulated by increased production of the short chain fatty acid, propionate, in the lumen of the gut to act on fatty acid receptor 2 [55]. Results from the large randomized controlled trial, DIOGENES, support an association between GLP-1, increased dietary fiber and better maintenance of weight loss [56]. Fasting CCK has been shown to respond to low compared to high fat diets [57] as well as other nutrients. The absence of a CCK response in this study may be because fat was held constant at 30% in each of the 10%, 15% and 25% protein diets.

This study suggests there may be a potential role for FGF-21 in driving increases in energy intake when the proportion of protein in the diet is reduced under controlled *ad libitum* study conditions. Changes in FGF-21 as a result of protein restriction may have consequences not only for total energy intake but also for body weight and metabolic health.

## Supporting Information

S1 File Study Protocol. (PDF)

S2 File CONSORT Checklist. (DOC)
