# Supplementary material for: Raised FGF-21 and Triglycerides Accompany Increased Energy Intake Driven by Protein Leverage in Lean, Healthy Individuals: A Randomised Trial
Source: PLoS One. 2016 Aug 18;11(8):e0161003. doi: 10.1371/journal.pone.0161003 (PMC4990330; doi:10.1371/journal.pone.0161003)
Supplement: S1 File — (PDF) [file pone.0161003.s001.pdf]

SECTION 9: DESCRIPTION OF PROJECT

Part A: Describe the study's background and scientific basis, aims, hypotheses and potential significance in LAY LANGUAGE (one page, including key references)*.

The aim is to test in controlled experiments the predictions of the 'protein leverage hypothesis' (PLH) for human obesity: the idea that food consumption in humans, like other animals, is adjusted to maintain a target protein intake (Simpson & Raubenheimer, 2005).

According to the PLH, the consumption of a low % protein diet typical of many Westernised countries inevitably requires the ingestion of additional energy. Conversely, the consumption of a diet that is relatively high in its proportion of protein requires the ingestion of lower levels of energy, creating the potential for weight loss.

The public health implications are substantial and there is a pressing need to test the PLH in tightly controlled experiments on lean and obese subjects. This project will provide such a test, and meet the following goals:

Primary goal: To investigate whether a change in the proportion of dietary protein alters total energy intake during ad libitum feeding on covertly manipulated diets.

Secondary goals: i) To identify whether there is a change in 24-hour blood and urine chemistry when the proportion of dietary protein is altered; ii) to explore whether there is a different response to protein manipulation in lean and obese subjects, as predicted by the PLH.

To test the PLH in humans, we will manipulate the protein to energy ratio of the diet for 4 consecutive days on 3 occasions while allowing subjects to eat ad libitum. If small changes in the proportion of protein in the diet impact on total energy intake there will be significant implications for weight control strategies. A 4-day treatment period has been chosen on the basis of data from Simpson et al. (18) and Weigle et al. (14), which indicate that stable patterns of intake in response to altered % dietary protein are apparent within 1-2 days, and persist over at least 70 days thereafter (14).

Pilot Study

Covertly manipulated diets are used to test the effects of changes in macronutrient composition and/or energy density on energy intake in humans. These diets are not usually tested simultaneously and so it is difficult to determine if the final result is due to the intended manipulation or due to changes that have taken place due to the nature of the manipulation. These changes may include differences in appearance, smell, taste and texture and can potentially affect the pleasantness of the food (Stubbs RJ 2001). Since pleasantness may influence amount eaten it is important that we ensure that our study foods, manipulated in macronutrient content are as similar as possible in pleasantness rating.

The primary aim of the pilot study is to determine if there is any difference in the pleasantness between the 10, 15 and 25% versions of a representative sample of test foods and if any such difference is due to the appearance, smell, taste and/or texture of the food. The secondary aim is to determine if the participants are able to determine the nutrient manipulation.

***You must satisfy the HREC that the study is valid and in accordance with accepted principles governing research involving humans. Parts A and B must be no longer than 2 pages and must be in a font size of at least 10 points.**

Part B: **Describe in LAY LANGUAGE the study procedures and their implications for the participants. Include discussion of the recruitment process, the inclusion/exclusion criteria, the research interventions, risks and side effects. Attach relevant technical information / documents (see checklist)***

Subjects 24 lean and 24 obese subjects, comprising equal numbers of men and women, will be analysed as separate groups. This sample size calculation is based on Stubbs et al (38), where 6 men were investigated with 7 days of indirect calorimetry and ad libitum food intake on three occasions.

Inclusion Criteria: Female or Male, 18-65 years; lean subjects, BMI <25kg/m^2; obese subjects, BMI 30-40 kg/m^2. Exclusion Criteria: Pregnancy or planning pregnancy, breastfeeding, known diabetes, known unstable or untreated elevated blood pressure or cholesterol, cardiovascular disease, chronic inflammatory conditions, medications that may interfere with glucose metabolism, smoking, alcohol consumption above current NHMRC guidelines, allergy or intolerance to any of the intervention foods, irregular eating patterns or eating disorder, following a weight reducing diet. Drop out: We estimate a 20% dropout rate and will increase our sample sizes accordingly.

After a screening interview and completion of a questionnaire, participants will attend the metabolic unit for an initial day of investigations and then three 4-day periods of dietary manipulation with 3 weeks between each visit. We will run experiments 2 weeks out of every 3. During each experiment week participants will be provided with ad libitum food comprised of 10, 15 or 25% protein. For the 4 day periods participants will live in a nearby rented unit (Woolcock Sleep Study Centre or Sydney University Village). Subjects will be supervised during the day. A surveillance system will be placed at the external doors of the apartment to monitor compliance during the night.

Subjects will be tested in single-sex groups of three, with all 3 subjects being lean or obese and all on the same dietary treatment (high, medium or low %P) per experimental week. The group will stay together throughout the full experiment, experiencing all 3 diet treatments. Across the three experimental weeks, each individual will be subjected to all three diets in different orders. Subjects will be age-matched between BMI categories.

Initial investigation day

Participants will arrive at the metabolic unit after an overnight fast, bringing a 4-day food diary and 24-h urine sample. Questionnaires will be completed to assess medical history, eating behaviour and physical activity. Measures will be taken of height, weight, waist circumference, body composition by bioelectrical impedance analysis (BIA), and blood pressure. Basal Metabolic Rate will be measured using indirect calorimetry.

Experiment week (repeated on 3 occasions with at least 2 weeks intervening between test periods.)

Day 1: Participants (lean or obese) will arrive at the Metabolic Unit from home after an overnight fast. Weight, waist circumference, BIA, blood pressure will be measured and a whole body DXA scan performed. Blood samples will be taken for biochemical analysis. Food will be provided ad libitum in excess and subjects invited to eat throughout the day. They will spend an hour in the afternoon on a supervised walk. At other times they will be confined to the Metabolic Unit or the Woolcock Sleep Study Centre / Sydney University Village apartment.

Days 2 and 3: Participants will arrive at the Metabolic Unit from the Woolcock Sleep Study Centre / Sydney University Village apartment Weight will be measured. Food will be provided ad libitum in excess and subjects invited to eat throughout the day. A continuous glucose monitor will be inserted before you leave the metabolic unit and worn until day 5. The glucose sensor is placed in the soft tissue of the upper outer hip with an injector device (larger, but similar to the device used to prick fingers when people with diabetes do blood glucose monitoring). The needle (similar in length and size to an insulin injection) is withdrawn leaving a short 2.5cm silastic cannula in the subcutaneous tissue. The sensor has attached adhesive strips which hod it in place and the monitor, which is worn on the belt, is attached to the sensor with a short cable. The only adverse events may be some bruising or bleeding at the site of injection or in rare cases, a local allergic response to the adhesive.

Day 4: Participants will arrive at the Metabolic Unit from the Woolcock Sleep Study Centre / Sydney University Village apartment. Weight will be measured. Participants will begin a 24 h urine collection. Visual analogue scales (VASs) for the measurement of subjective appetite for sweet and savoury foods will be completed hourly throughout the day. Food and exercise as day 2 and 3.

Day 5: Participants will arrive at the Metabolic Unit from the Sleep Study Centre / Sydney University Village apartment. The 24 h urine collection will be completed. Basic measures, whole body DXA and blood samples will be performed as on day 1. The continuous glucose monitor will be removed. Subjects will be offered a free choice breakfast.

Pilot Study: 20 healthy subjects aged 18-40 with a BMI < 25kg/m^2 (see above for exclusion criteria). Participants will be invited to the Human Nutrition Unit for a 1 hour taste testing session at midday after a 4 hour fast (ie between breakfast and taste testing). The test foods will be a representative selection of the foods to be used in the approved study. Participants will be presented with approximately 50g of the 10, 15 and 25% versions of the test foods simultaneously. Participants will then be asked to complete 3 questionnaires to test for changes in palatability, sensory components and nutrient composition.
